# Timing of maternal vaccination against COVID-19 for effective protection of neonates: cohort study

**DOI:** 10.3389/fimmu.2024.1359209

**Published:** 2024-07-08

**Authors:** Aleksandra Nowakowska, Seung Mi Lee, Minjee Kim, Jungmin Chun, Sehyun Kim, Byung Chul Kim, Hyun Ju In, Eunji Lee, Chanyeong Lee, Hyeondong Lee, Yuyeon Jang, Hansam Cho, Jinha Kim, Jeesun Lee, Hee-Jung Lee, Yoo-Kyoung Lee, Joong Shin Park, Young Bong Kim

**Affiliations:** ^1^ Department of Biomedical Science and Engineering, Konkuk University, Seoul, Republic of Korea; ^2^ Department of Obstetrics and Gynecology, Seoul National University College of Medicine, Seoul, Republic of Korea; ^3^ KR Biotech Co., Ltd, Seoul, Republic of Korea; ^4^ Korea Disease Control and Prevention Agency, National Institute of Health, National Institute of Infectious Diseases, Center for Vaccine Research, Division of Vaccine Development Coordination, Cheongju, Republic of Korea; ^5^ Department of Bio-industrial Technologies, Konkuk University, Seoul, Republic of Korea

**Keywords:** SARS-CoV-2, maternal vaccination, pregnancy, COVID-19, passive immunity

## Abstract

**Introduction:**

Although the safety and effectiveness of COVID-19 vaccination during pregnancy have been proven, there is still little data explaining neonatal outcomes of maternal pre-pregnancy vaccination.

**Methods:**

Here, we investigated the impact of vaccination and SARS-CoV-2 infection on maternal-neonate immune response in a cohort study involving 141 pregnant individuals, and defined the importance of maternal COVID-19 vaccination timing for its effectiveness.

**Results and discussion:**

Our data indicate that vertically transferred maternal hybrid immunity provides significantly better antiviral protection for a neonate than either maternal post-infection or post-vaccination immunity alone. Higher neutralization potency among mothers immunized before pregnancy and their newborns highlights the promising role of pre-pregnancy vaccination in neonatal protection. A comparison of neutralizing antibody titers calculated for each dyad suggests that infection and pre-/during-pregnancy vaccination all support transplacental transfer, providing the offspring with strong passive immunity against SARS-CoV-2. Analysis of neutralizing antibody levels in maternal sera collected during pregnancy and later during delivery shows that immunization may exert a positive effect on maternal protection.

## 1 Introduction

Pregnancy is a challenging time for the human body. The maternal immune system must maintain sufficient immunity to protect mother and baby while suppressing the natural defense against an allogeneic fetus; it thus undergoes many adaptations that make pregnant individuals more susceptible to pathogens (1, 2) Additionally, psychological and endocrinological changes, such as reduced lung capacity due to uterine expansion, affect pregnancy-associated homeostasis and render the body particularly sensitive to respiratory infection, including that by SARS-CoV-2 (3, 4). Infection may have unfavorable fetal effects, such as preterm birth, stillbirth, miscarriage, and severe illness in infants. Several studies found that placenta tissue, umbilical cord, and amniotic fluid samples can test positive at the time of birth. The presence of SARS-CoV-2 in fetal and maternal tissue indicates that *in utero* vertical transmission of the virus could pose a threat to the newborn (2, 3, 5). On the other hand, passive transfer of SARS-CoV-2-specific antibodies acquired by mothers after infection may provide offspring with protection against coronavirus (6, 7).

Given that the mentioned passive immunity is provided to newborns by mothers and vaccination increases the maternal antibody concentration leading to enhanced neutralizing antibody (nAb) transfer, the benefits of prenatal COVID-19 vaccination should be seriously considered. Despite these positive outcomes of maternal immunization, however, pregnant and lactating individuals were excluded from all initial COVID-19 vaccine trials (8). Fortunately, subsequent studies supported the safety and immunogenicity of COVID-19 vaccines in mothers and their delivered infants and confirmed the meaningful role of maternal vaccination (9–11). Recent guidelines of the Center for Disease Control and Prevention (CDC), American Congress of Obstetricians and Gynecologists (ACOG), and National Institutes of Health (NIH) highly recommend maternal COVID-19 vaccination (10, 12, 13).

In this cohort study, we defined nAb ND50 titers against SARS-CoV-2 Wuhan-Hu-1 and Omicron BA.5 variants in serum samples collected from 141 mothers and their newborns at Seoul National University Hospital in South Korea. We examined the impact of infection and the timing of maternal COVID-19 vaccination on the maternal-newborn immune response.

## 2 Materials and methods

### 2.1 Study design and clinical sample collection

Pregnant women admitted to the Seoul National University Hospital were selected to participate in this study. Qualified participants were at least 18 years old and provided their informed consent to participate in the research. The statuses of pregnancy, COVID-19 vaccinations, and infection were monitored on an ongoing basis until delivery. Vaccination status before and during pregnancy was confirmed based on the Vaccination Certificate System established by the Korea Disease Control and Prevention Agency. The absence of pre-pregnancy SARS-CoV-2 infection was defined based on self-reports and medical history of participants. Infection or its lack during pregnancy was laboratory-confirmed. Blood samples were drawn from participants at the time of enrollment during pregnancy. Later, maternal and newborn blood samples were collected also at the time of delivery.

### 2.2 Ethic approval

The institutional review board of Seoul National University Hospital approved this study (No 2112-109-1284).

### 2.3 Participants’ characteristics

A total of 141 mother-newborn dyads were involved in our study (**Table 1**). According to the time of vaccination and SARS-CoV-2 infection, pregnant individuals were divided into the following six groups: vaccination-only (before or during pregnancy), naïve infection (infection without previous vaccination), and breakthrough infection (infection after vaccination which was administered either before or during pregnancy), and control (neither infection nor vaccination).

**Table 1:** Number of study participants in each group.

	Unvaccinated	Vaccinated	
		Before pregnancy	During pregnancy
Infected	20 (14%)	31 (22%)	31 (22%)
Non-infected	8 (6%)	29 (21%)	22 (16%)

### 2.4 Cells and viruses

African green monkey Vero E6 cells (ATCC, USA) were cultured in DMEM (HyClone, USA), supplemented with 10% FBS and 1% penicillin-streptomycin (Gibco BRL, USA). SARS-CoV-2 Wuhan-Hu-1 (βCoV/Korea/KCDC03/2020 NCCP No.43326) and Omicron BA.5 (GRA: BA.5 NCCP No.43426) variants were obtained from the National Culture Collection for Pathogens (Korea Disease Control and Prevention Agency). All experiments were performed in the Biosafety Level 3 facility of Konkuk University.

### 2.5 Plaque reduction neutralizing assay

Briefly, each 2–fold dilution of heat-inactivated serum was mixed with an equal volume containing diluted virus (50 PFU/0.1 ml) and the mixture was incubated for 1 hour at 37°C. Next, confluent Vero E6 cells prepared on 24–well plates were inoculated with the serum/virus mixture and incubated at 37°C for 1 hour. The mixture was removed and the cell monolayer was overlaid with DMEM containing 1% low melting temperature agarose (Lonza, USA) and 5% FBS. After 3 days of incubation, the plates were stained with a crystal violet staining solution and visible plaques were counted. The 50% neutralizing dose (ND50), which was defined as the highest serum dilution that caused a >50% reduction in the number of plaques, was calculated using the Karber formula: log10ND50=m-Δ(Σp-0.5).

### 2.6 Quantification and statistical analysis

Data presented on dot and violin plots include medians ± maximum/minimum values, while box plots present the nAb transfer ratio (TR) with median, 25th and 75th percentiles, and maximum/minimum values. Relations of paired variables are represented by symbol plots and differences plots. nAb TRs were calculated by the formula: infant ND50 (log10)/mother ND50 (log10). All statistical analyses and data plotting were performed with GraphPad Prism 8 (GraphPad Software, USA). For comparison of multiple groups, we used the Kruskal-Wallis test with Dunn’s multiple comparison tests. Statistical significance between two groups was determined by two-tailed Mann-Whitney test or two-tailed Wilcoxon signed-rank test for paired data. P < 0.05 was considered statistically significant and is marked on figures as: * (p < 0.05); ** (p < 0.01); *** (p < 0.001); ****(p < 0.0001).

## 3 Results

### 3.1 Effectiveness of hybrid immunity

**Figure 1:**
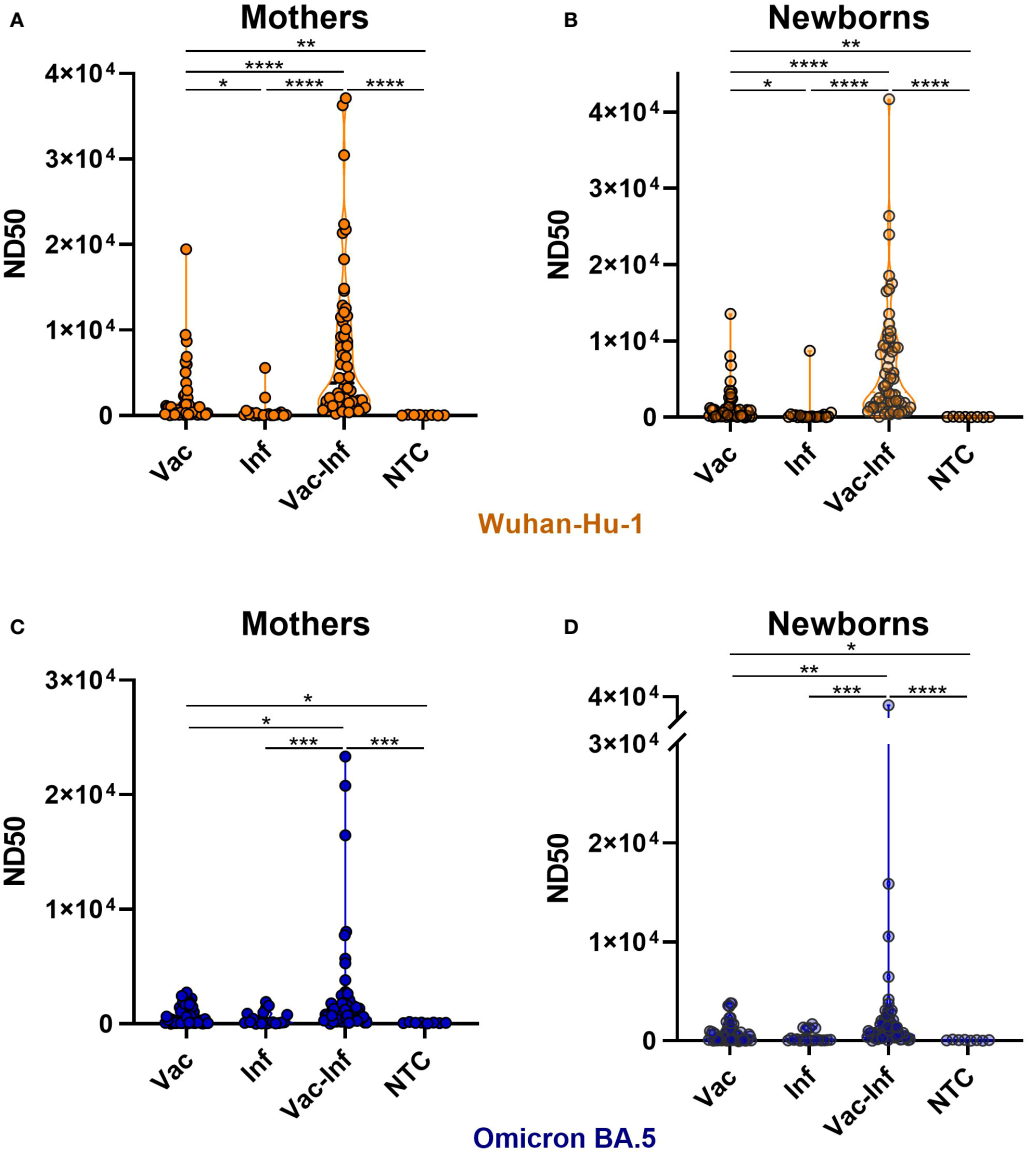
Impact of vaccination and infection on serum neutralizing activity. Serum samples were collected from mothers **(A, C)** and newborns **(B, D)** at the time of delivery. ND50 titers against SARS-CoV-2 Wuhan-Hu-1 **(A, B)** and Omicron BA.5 **(C, D)** strains were calculated independently. The violin plots represent the distribution of ND50 values in each group. Statistically significant differences between groups were defined based on the Kruskal-Wallis test with Dunn’s multiple comparisons tests. Vac vaccination-only (before or during pregnancy), Inf naïve infection (infection without previous vaccination), Vac-Inf breakthrough infection (infection after vaccination administered either before or during pregnancy), NTC (neither infection nor vaccination) *(p < 0.05); **(p < 0.01); *** (p < 0.001).

Analysis of maternal and neonatal serum-neutralizing activity against SARS-CoV-2 variants identified the Wuhan strain as being highly susceptible to nAbs produced in vaccinated and later infected (Vac-Inf) individuals. The ND50 median value for Vac-Inf maternal and neonatal samples (mothers 3819, [1476–10330]; neonates 3282, [1355–9195]) was about 6–fold and 32–fold higher (**Figure 1A, B**) than those in the vaccinated-only Vac (mothers 658.4, [152.1–2068], neonates 649.7, [149.9–1236]) and infected-only Inf (mothers 119.3, [58.7–324.1]; neonates 100.8, [52.05–262.9]) groups, respectively. Comparison of the median values between the vaccinated-only Vac and infected-only Inf groups suggests that vaccination is a better immunity booster than previous infection.

**Figure 2:**
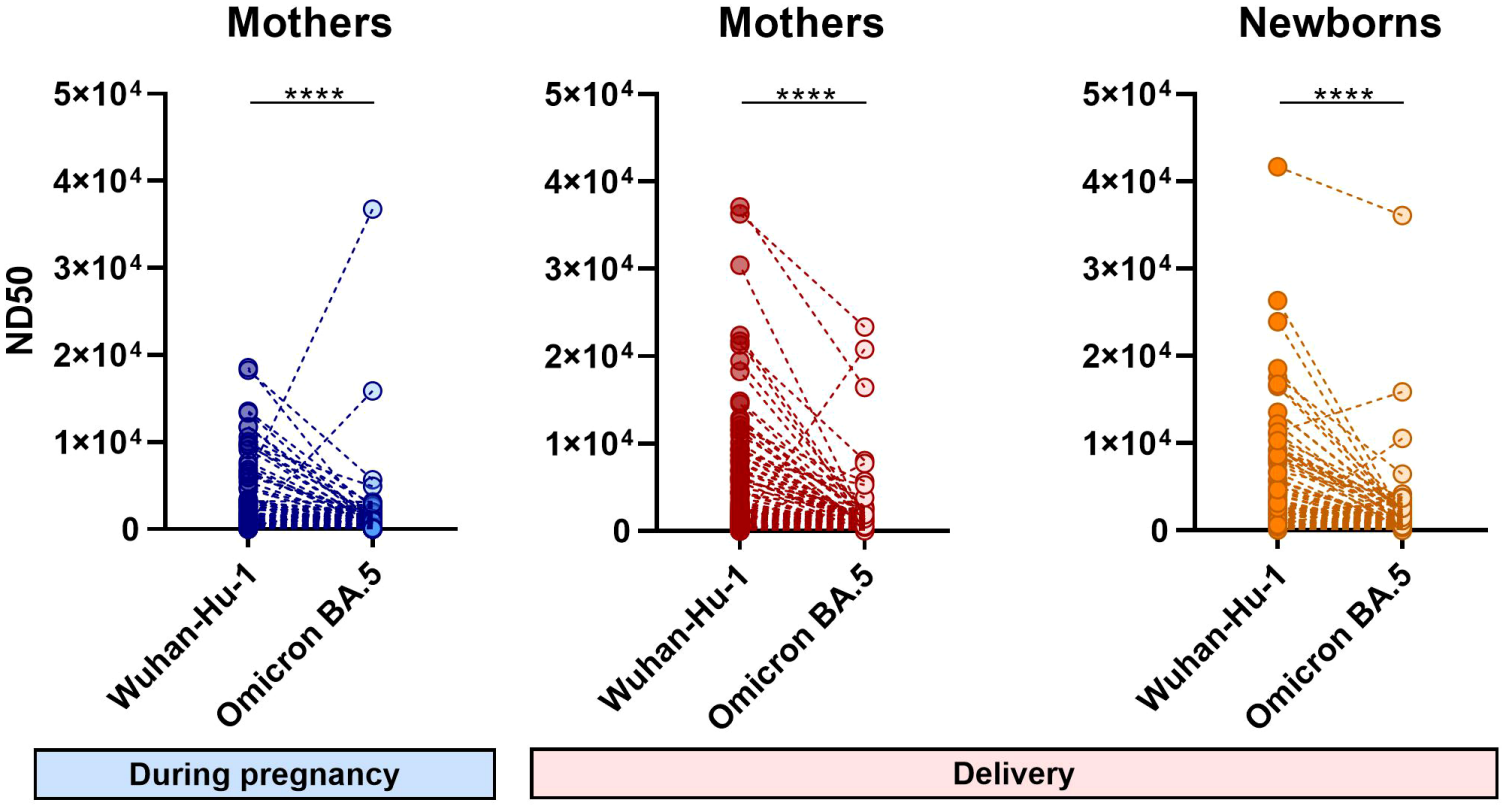
Differences in the 50% neutralizing dose values of individual samples for Wuhan-Hu-1 and Omicron BA.5 strains. Symbol plots present the ND50 values of each sample calculated independently for different virus strains. Significant differences were determined by Wilcoxon signed-rank test for paired data. N=141 ****(p < 0.0001).

The neutralizing activity against Omicron was noticeably lower than that against Wuhan (**Figures 1A-D**, **2**). For Omicron, the neutralizing potencies in Vac-Inf mothers (1072, [424.5–1804]) and infants (884.8 [444.3–1905]) were significantly higher than those in the corresponding Vac (mothers 361.3, [95.1–1542]; neonates 394, [78.7–1102]) and Inf (mothers 137.2, [71.4–862.5]; neonates 97.2, [47.7–968.1]) groups. The neutralizing protection levels were fairly similar in the Vac and Inf groups (**Figures 1C, D**), but the ND50 values obtained for the Vac groups were significantly higher than those obtained for the control NTC groups.

### 3.2 The impact of maternal vaccination timing on the neutralizing antibodies activity

**Figure 3:**
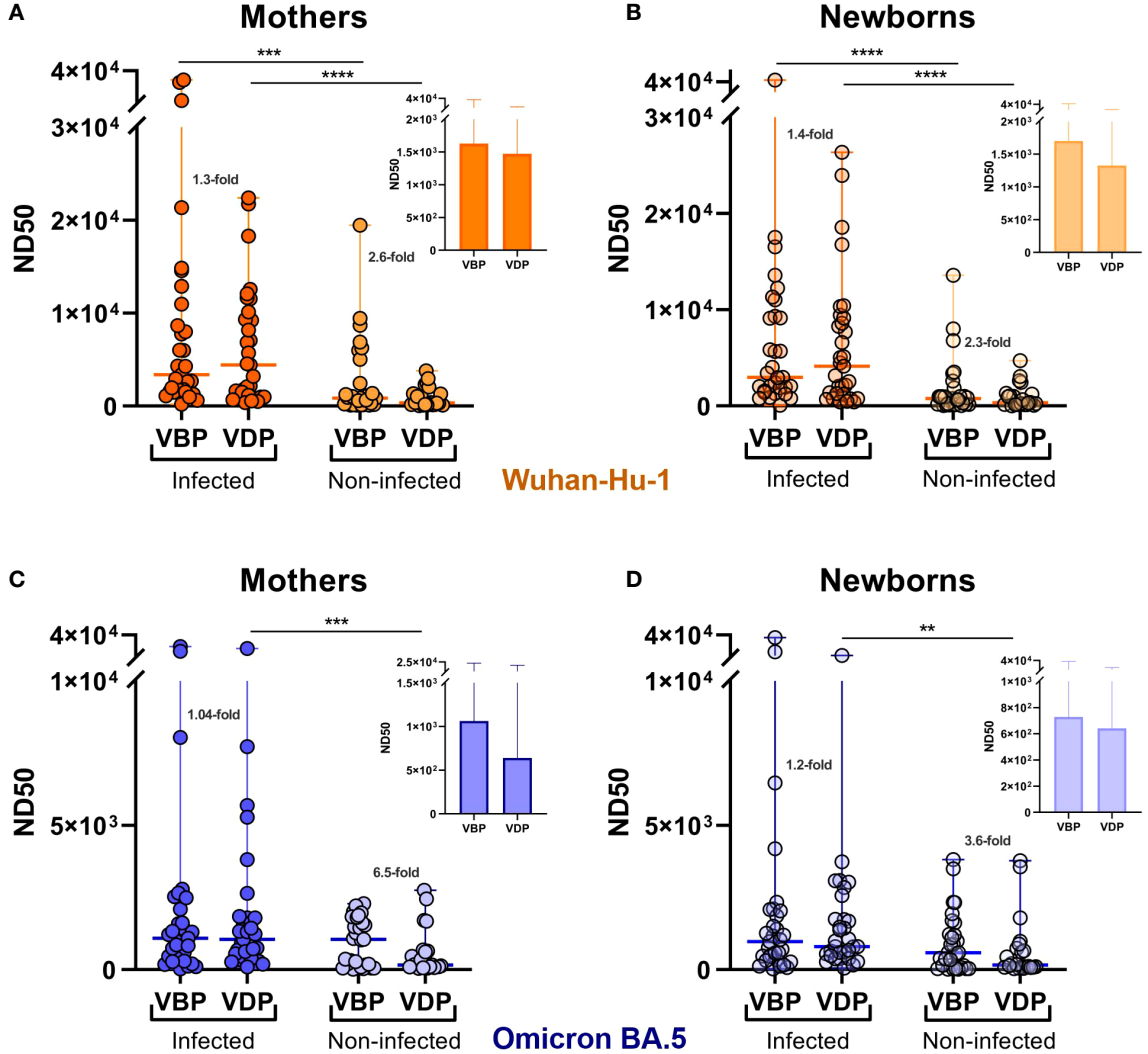
Effect of infection and vaccination timing on the serum neutralizing activity against SARS-CoV-2. Serum samples were collected from the mothers **(A, C)** and newborns **(B, D)** at the time of delivery. ND50 values were calculated for SARS-CoV-2 Wuhan-Hu-1 **(A, B)** and Omicron BA.5 **(C, D)** strains. Dot plots represent a distribution of ND50 values with median and range values for each group. Statistical significance between subgroups VBP and VDP within the infected or non-infected groups was determined by two-tailed Mann-Whitney test. Statistical significant differences between infected and non-infected groups were defined based on Kruskal-Wallis test with Dunn’s multiple comparisons tests. VBP, Vaccination Before Pregnancy; VDP, Vaccination During Pregnancy. **(p < 0.01); ***(p < 0.001); ****(p < 0.0001).

Analysis of ND50 values divided into groups vaccinated before pregnancy (VBP) or during pregnancy (VDP) revealed that vaccination timing did not have a major effect on the nAb level in the infected cohort. Compared to the corresponding VBP groups, the median ND50 values for the VDP groups (mothers 4429, [1060–10114]; neonates 4136, [1229–9134]) against Wuhan (**Figures 3A, B**) were 1.3–fold greater for maternal samples (VBP 3364, [1548–10979]) and 1.4–fold greater for neonatal samples (VBP 2960, [1546–9322]). For Omicron, in contrast, the same ratios of ND50 median values were close to 1 (**Figures 3C, D**). Noticeable but non-significant differences were observed in the proportions of neutralizing antibodies for the non-infected cohort. Regarding vaccination timing, pre-pregnancy vaccination more effectively triggered neutralizing activity against both virus strains in maternal and infant sera. The median ND50 values against Wuhan for mothers and neonates of the VBP group (mothers 865.1, [195.4–3746]; babies 769.9, [175.5–1425] were 2.6–fold and 2.3–fold higher, respectively, than those of the VDP group (mothers 337.5, [146.1–1509]; babies 338, [139.6–1259]), while those against Omicron (mothers 1051, [132.1–1716]; babies 586.9, [53.6–1424]) were 6.5–fold (mothers 161.1, [89.9–649.2]) and 3.6–fold (babies 162.2, [84.5–774.9]) higher, respectively.

### 3.3 Vertical transfer of maternal SARS-CoV-2 neutralizing antibodies

**Figure 4:**
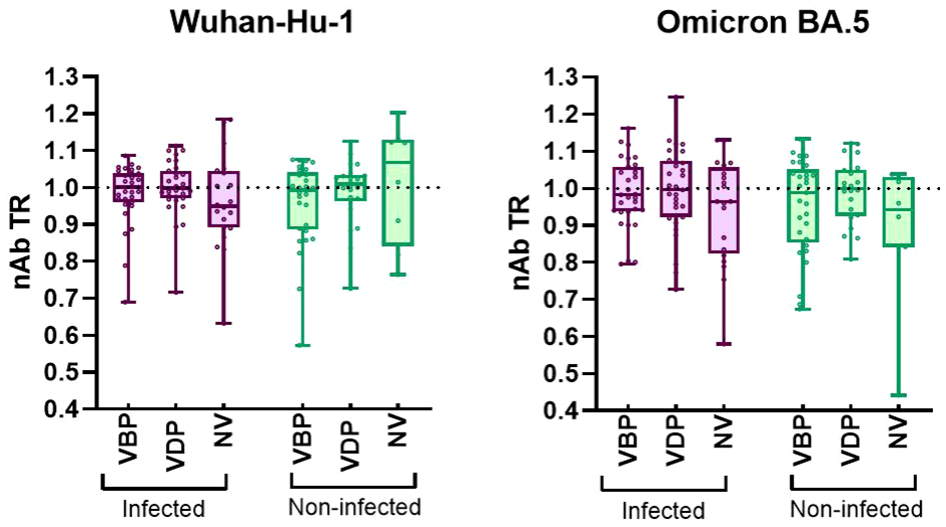
Transplacental transfer ratio of nAbs against SARS-CoV-2 Wuhan-Hu-1 and Omicron BA.5. A The transfer ratio was calculated for each mother-newborn dyad as (log10ND50 Baby)/(log10ND50 Mother). The box plots represent the nAb transfer ratio (nAb TR) efficiency. Plots include the median, 25th and 75th percentiles, and maximum/minimum values for each analyzed group. Statistical significance between VBP or VDP or NV subgroups from infected and non-infected groups was determined by two-tailed Mann-Whitney test. Statistical significant difference between subgroups VBP, VDP and NV within the infected or non-infected groups was determined by Kruskal-Wallis test with Dunn’s multiple comparisons tests. No significant difference was found. VBP, Vaccination Before Pregnancy; VDP, Vaccination During Pregnancy; NV, Non-vaccination.

High efficiency of *in utero* vertical transfer was observed in the vaccinated groups regardless of immunization timing (**Figure 4**). A slightly decreased TR was noted in the naïve infected cohort, where the median was 0.95 [0.89–1.04] for Wuhan and 0.96 [0.82–1.06] for Omicron. Despite the lack of significant differences among all groups, TR distribution analysis showed that most of the variables from the infected cohort had higher values in the vaccinated group than in the unvaccinated group. There was no difference among the median TR values for results ordered by infection status. The lowest antibody TR should have been found in the unvaccinated-uninfected cohort, but the median value in this group was 1.07 [0.84–1.13] for Wuhan and 0.94 [0.84–1.03] for Omicron. Surprisingly, 1.07 was the highest median value obtained for this parameter. However, this is probably due to there being too few variables in the distribution, rather than reflecting biological factors.

A strong correlation between maternal antibodies and passively acquired neonatal antibodies was also confirmed by comparative analysis of ND50 values (**Supplementary Figure S1**). There was no significant difference between matched mother-offspring serum ND50 titers, regardless of the analyzed group or virus variant. A few newborn serum ND50 values were outliers relative to the maternal ND50 values, likely reflecting individual characteristics of mothers and offspring and/or that the TR might assume an erroneously high value if the maternal antibody level is low.

### 3.4 Dynamic of SARS-CoV-2 neutralizing antibodies during pregnancy

**Figure 5:**
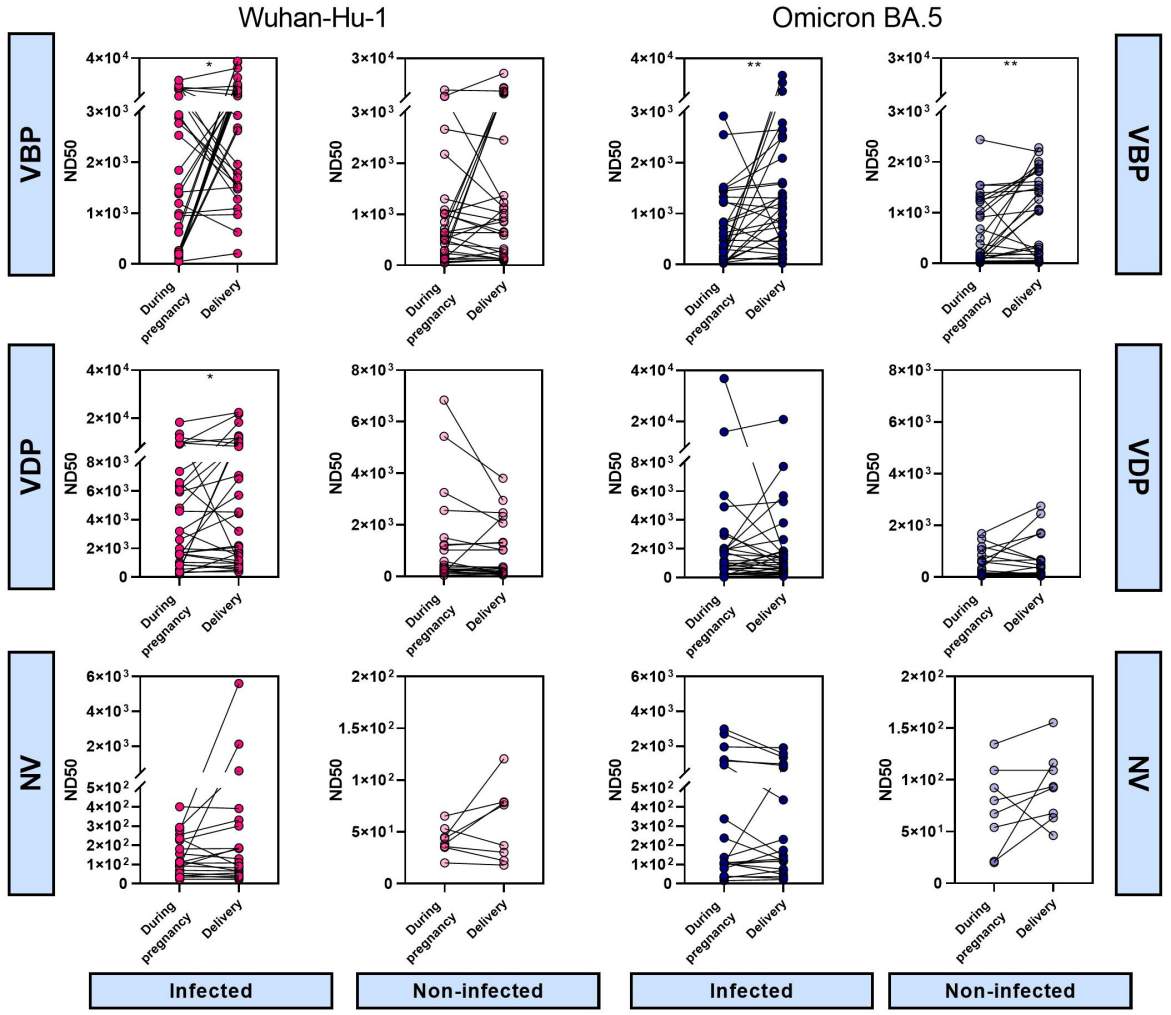
Vaccination- and infection-induced differences in neutralizing activity over time. The 50% neutralizing dose against Wuhan-Hu-1 and Omicron BA.5 strains were defined for paired maternal samples collected at different times of pregnancy. Symbol plots represent the relationship between sampling time and ND50 values calculated for each matched sample pair. Significant differences were determined by Wilcoxon signed-rank test for paired data. VBP, Vaccination Before Pregnancy; VDP, Vaccination During Pregnancy; NV, Non-vaccination.

Analysis of changes in maternal ND50 values during pregnancy indicated that, in the VBP group, the neutralizing potency tended to increase or stay the same during pregnancy in both infected and non-infected cohorts. Thus, regardless of infection, vaccination before pregnancy helped mothers maintain nAb protection until delivery (**Figure 5**, **Supplementary Figure S2**, first panel). In the VDP group, the same pattern in ND50 changes during pregnancy applied to both Wuhan and Omicron strains, regardless of infection status (**Figure 5**, **Supplementary Figure S2**, second panel). Here, we observed an upward trend across groups, indicating that maternal vaccination increased the neutralizing protection against SARS-CoV-2. In all unvaccinated groups, maternal neutralizing immunity remained at the same low level during pregnancy (**Figure 5**, **Supplementary Figure S2**, third panel).

### 3.5 Differences in neutralizing activity against Wuhan-Hu-1 and Omicron BA.5 strains

The differences in neutralizing activity against Wuhan and Omicron calculated for the same serum sample were highly significant (**Figure 2**). Most sera showed much less neutralization activity against the Omicron BA.5 variant. The few cases that showed much higher neutralizing activity against Omicron BA.5 might have been previously infected with an Omicron variant.

### 3.6 Study limitations

Our study has several limitations. First, we did not obtain detailed data for the study population, such as exact maternal age, fetus sex, medical history, or information about the severity of infection in positive-confirmed cases. All these factors could affect maternal and offspring immunity against viruses. Next, our analysis of the immunized cohort was based on the last received vaccination date. A full history of immunization containing information about the vaccine type and number of doses would allow for a deeper analysis of vaccination outcomes in relation to pregnancy. The biggest limitation was that we lacked a precise analysis of the exact period between collections of the two maternal blood samples used in our examination of neutralizing potency variation over pregnancy. We were also unable to put the infection on the timeline or distinguish the trimester of vaccination, which greatly generalized our considerations. Finally, the sample sizes differed between the analyzed groups and the control group (unvaccinated-uninfected) contained only eight mother-infant pairs. The use of a larger and more equally distributed sample could increase the statistical significance and reveal additional differences between the analyzed groups.

## 4 Discussion

Here, we aimed to understand the relationship between maternal and newborn immune responses to COVID-19 vaccination and previous infection by analyzing the neutralizing activity levels in serum samples. Our results indicate that maternal COVID-19 immunization tends to offer more reliable protection than natural immunity acquired after infection. Furthermore, mothers and newborns from the vaccinated and later infected group had the highest concentration of neutralizing antibodies, compared to those with vaccination or natural immunity alone, which is consistent with publications explaining that repeated triggering of adaptive immunity by vaccination and infection prompts antibody production together with improving memory B cell response, and T cell activation (14, 15). Recorded in our study a higher level of antibodies might indicate that hybrid immunity is more effective than maternal postvaccination-only immunity or postinfection-only immunity; however, to draw such a conclusion, it would be necessary to examine also virus protein-specific antibody response, T- and B-cells response and level of cytokines in serum. In addition, higher levels of neutralizing antibodies detected in the neonatal samples suggest that hybrid immunity provided by the mother may critically contribute to passively protecting newborns shortly after birth. Believing that hybrid immunity guarantees a combination of long-lasting and broad antiviral defenses (16, 17) and following the continuous increase in the total number of infected people across the World and the emergence of new virus variants including variants of concern (VOCs) (18, 19), the use of COVID-19 vaccines should be prioritized. Future studies seem to be essential for discovering the full potential of vaccination in providing hybrid immunity among populations in which the transmission of the virus is higher and among pregnant women at risk of severe disease (3, 4).

In the analysis of the vaccination timing, there was no significant difference between groups. This may reflect that we divided the time into before and during pregnancy without specifying the months before pregnancy or the trimester of pregnancy (20). Therefore, there is a possibility that our study included participants vaccinated a short time before conception who were assigned to VBP group and/or vaccinated shortly after conception who were assigned to VDP group. In those cases, the timing of the immunization would be very similar although assigned to two separate groups. Such generalization could blur significant differences across groups. Despite this, our results showed that the level of neutralizing serum antibodies was dependent on vaccination timing. Based on postvaccination antibody peak duration data (21, 22), it is expected that the shorter the period between the last vaccination and sample collection, the greater the antibody activity. However, in the infected cohort, there were no noticeable differences between the VBP (longest period) and VDP (shortest period) groups for both tested viral strains. We speculate that the infection experienced in a similar period of time by the study participants equalized the number of antibodies, obscuring the impact of the vaccination time itself on these results. Interestingly, the non-infected group exhibited higher neutralizing titers in the VBP group; this suggests that, over time, the antibodies could decrease in quantity while increasing in quality. This would be consistent with the presence of affinity maturation, which is reflected by improved avidity, affinity, and neutralizing potency of antibodies due to molecular changes in B cells (23). Considering the immune instability seen during pregnancy and the maturation of lasting post-vaccination immunity (24, 25), we conclude that COVID-19 immunization received before pregnancy may have benefits comparable to prenatal immunization, however keeping in mind that our investigation focused mainly on the naturalization capacity of antibodies, further research including analysis of the efficiency and effectiveness of vaccines must be conducted to explain in detail the promising role of pre-pregnancy immunization against viral diseases and its real-world application. Additionally, knowing that the transfer of antibodies from mother to baby depends - among others - on gestational age (26), a detailed weekly analysis of the pre-/during-pregnancy vaccination outcomes could be done to determine the sufficient immunization timing which ensures the most effective transfer of antibodies before delivery and the best protection in cases of mother infection during pregnancy.

Our analyses of data distribution and the interquartile ranges of transplacental transfer efficiency suggested that mother-to-baby antibody transmission was intensified in those with vaccination, and vaccination-infection, indicating that hybrid immunity and vaccination itself may play supporting roles. This was further supported by the reduced transmission ratio observed in the unvaccinated-infected group. The median TR values in all vaccinated groups were approximately 1, which agrees with data reported for maternal influenza and Dtap vaccinations (27, 28). Only a few cases exhibited lower TRs in the vaccinated group, and there was no apparent relationship between vaccination before or during pregnancy and the level of antibodies conveyed to newborns. Taken together, our results revealed that the efficiency of antibody transfer was ~100%, indicating that COVID-19 immunization and infection possibly provide newborns with passive immunity against SARS-CoV-2 prior to birth.

Comparison of neutralizing antibodies in maternal sera collected during pregnancy and later during delivery indicated that the protective effect of immunization before or during pregnancy increased over time or remained at the same level. Although the antibody level generally increased over time in the vaccinated groups, the levels decreased in a few isolated cases. Moreover, the changes in ND50 values between the two time points differed in magnitude within each group, especially where statistical significance was found. These dynamics of neutralizing antibodies could be influenced by infection history, vaccine type, booster number, and/or vaccination time (reflecting the time needed for antibodies to peak and mature), and maternal immunity fluctuation across trimesters. Together, our results suggested that vaccination stimulates the neutralizing potency of maternal antibodies during pregnancy, but the specific effects may be influenced by many factors and their complex relationships. Therefore, further study is needed to support the development of an unambiguous explanation for this issue.

Comparison of the ND50 between viruses showed that the neutralizing activity of the vaccine was much lower against Omicron BA.5, which is consistent with other studies proving weakened efficacy against Omicron subvariants (29–33). Our research was conducted around the time the Omicron BA.5 virus emerged in South Korea. Since newly emerging variants are more likely to escape from pre-exiting neutralizing antibodies (33), our results should come as no surprise. Unfortunately, we did not have information on which specific variant of the virus the study participants were infected with, so making a precise analysis of potential immunity evasion was impossible.

Regardless of the immune escape of Omicron variants (34, 35), COVID-19 immunization effectively protects against infection (36, 37), especially when the recently produced Omicron subvariant-targeting boosters are used (38). Discussing further, COVID-19 vaccination constitutes an important line of defense for pregnant women, among whom Omicron infection is associated with poor outcomes, especially in the unvaccinated population (39, 40). As reported to this day, vaccination received during pregnancy - also during the Omicron predominant period - increases the protection of infants aged 0 to 6 months, which is reflected in reduced risk of SARS-CoV-2 infection, infection-associated hospitalization, and morbidity (40–45). The effectiveness of such protection depends on the number of received vaccine doses, which was confirmed by the highest level of SARS-CoV-2-specific IgG antibodies in fully vaccinated pregnant and their offspring when compared to different groups of nonvaccinated, vaccinated, and infected parturient women (46). When discussing the importance of maternal vaccinations for the health of neonates, we should also remember about role of parental vaccinations in the indirect protection of newborns and older children sharing the same household (45, 47, 48).

In summary, we herein show that newborn and pregnant individuals can benefit from long-term immunity elicited by COVID-19 immunization before pregnancy. Since pregnant women and their offspring are vulnerable to a particularly severe course of SARS-CoV-2 infection and vaccinated women transmit passive immunity to their infants, we strongly recommend immunization for those who are trying to conceive and those who are pregnant. Since COVID-19 vaccine immunogenicity and efficacy are influenced by many factors, such as the vaccine platform, received number of doses, individual characteristics, and pre-existing immunity, the phenomena of maternal antibody response to vaccination require further investigations (49).

## Data availability statement

The original contributions presented in the study are included in the article/**Supplementary Material**. Further inquiries can be directed to the corresponding authors.

## Ethics statement

The studies involving humans were approved by The institutional review board of Seoul National University Hospital (No 2112-109-1284). The studies were conducted in accordance with the local legislation and institutional requirements. Written informed consent for participation in this study was provided by the participants’ legal guardians/next of kin.

## Author contributions

AN: Data curation, Formal analysis, Writing – original draft, Writing – review & editing, Investigation. SL: Data curation, Formal analysis, Writing – review & editing, Investigation. MK: Data curation, Formal analysis, Writing – review & editing. JC: Writing – review & editing, Investigation. SK: Investigation, Writing – review & editing. BK: Funding acquisition, Writing – review & editing. HI: Funding acquisition, Writing – review & editing. EL: Investigation, Writing – review & editing. CL: Investigation, Writing – review & editing. HL: Investigation, Writing – review & editing. YJ: Investigation, Writing – review & editing. HC: Investigation, Writing – review & editing. JK: Investigation, Writing – review & editing. JL: Writing – review & editing. HL: Data curation, Formal analysis, Writing – review & editing. YL: Funding acquisition, Writing – review & editing. JP: Conceptualization, Methodology, Project administration, Supervision, Writing – review & editing. YK: Conceptualization, Methodology, Project administration, Supervision, Writing – original draft.
