# Supplementary material for: Timing of maternal vaccination against COVID-19 for effective protection of neonates: cohort study
Source: Front Immunol. 2024 Jul 8;15:1359209. doi: 10.3389/fimmu.2024.1359209 (PMC11260787; doi:10.3389/fimmu.2024.1359209)
Supplement: Supplementary file 1 [file DataSheet_1.docx]

Supplementary Material

# Supplementary Figures

#
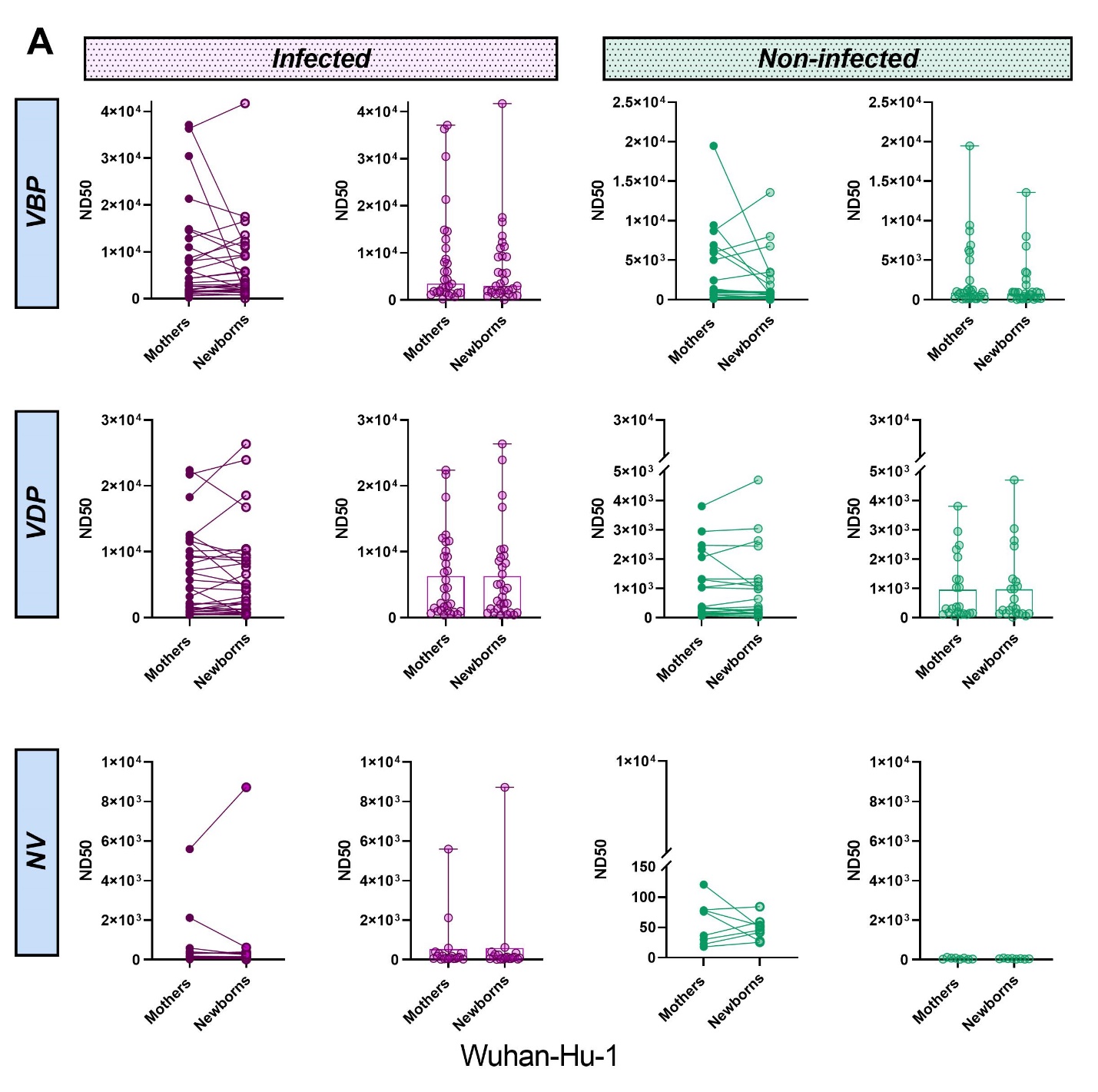


**
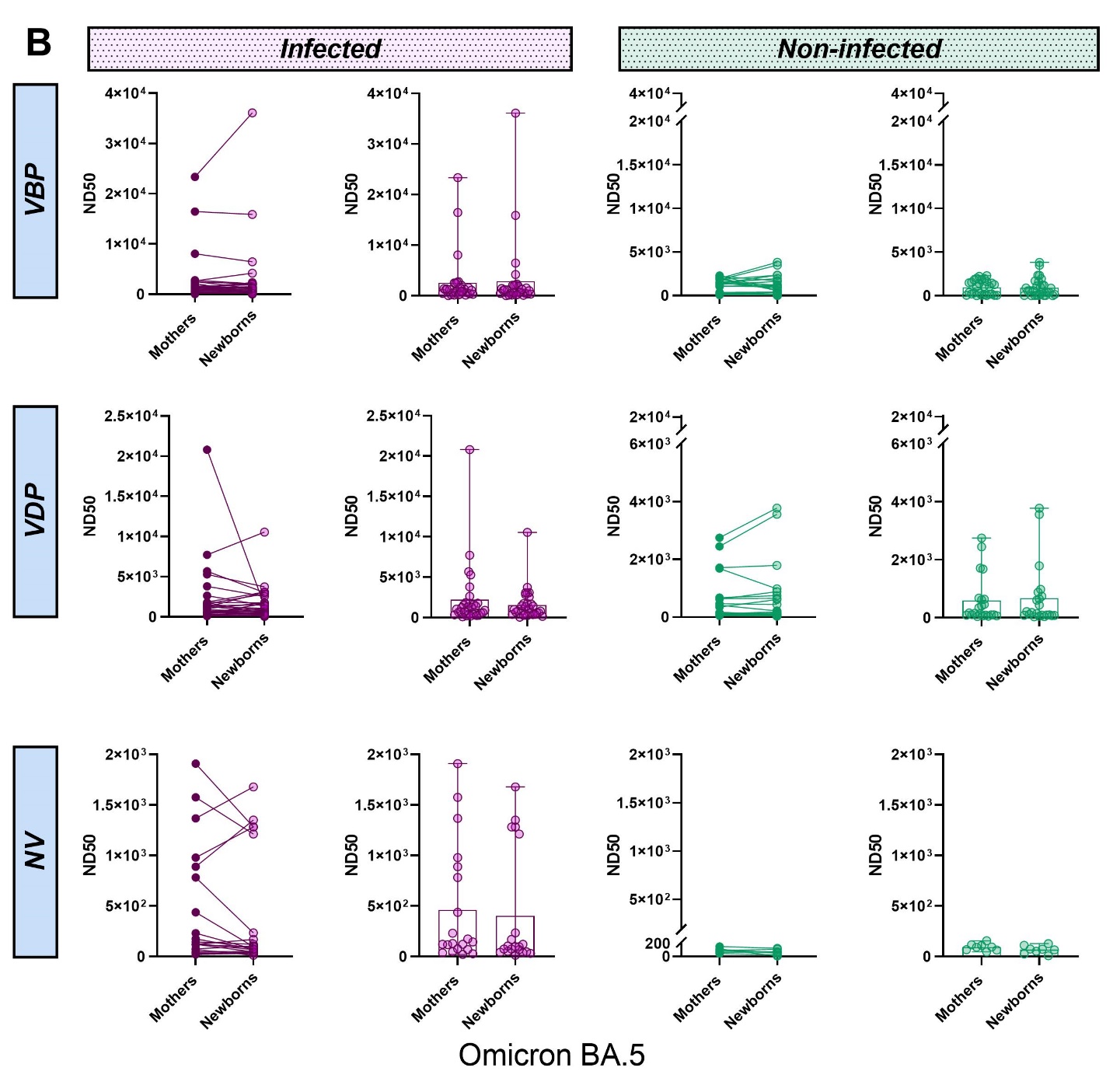
**

**Supplementary Figure 1.** Placental antibody transfer efficiency. Graphs show relations between neutralizing activity in maternal and newborn sera collected during delivery. ND50 values were calculated against Wuhan-Hu-1 (**A**) and Omicron BA.5 (**B**) strains. The dot plots present the distribution of ND50 values with median and range values for each group; symbol plots show connected ND50 values calculated for each mother-newborn dyad. There was no significant difference based on Wilcoxon signed-rank test for paired data. VBP Vaccination Before Pregnancy, VDP Vaccination During Pregnancy, NV Non-vaccination. VBP/Infected(N=31), VBP/Non-infected(N=29), VDP/Infected(N=31), VDP/Non-infected(N=22), NV/Infected(N=20), NV/Non-infected (N=8).

**
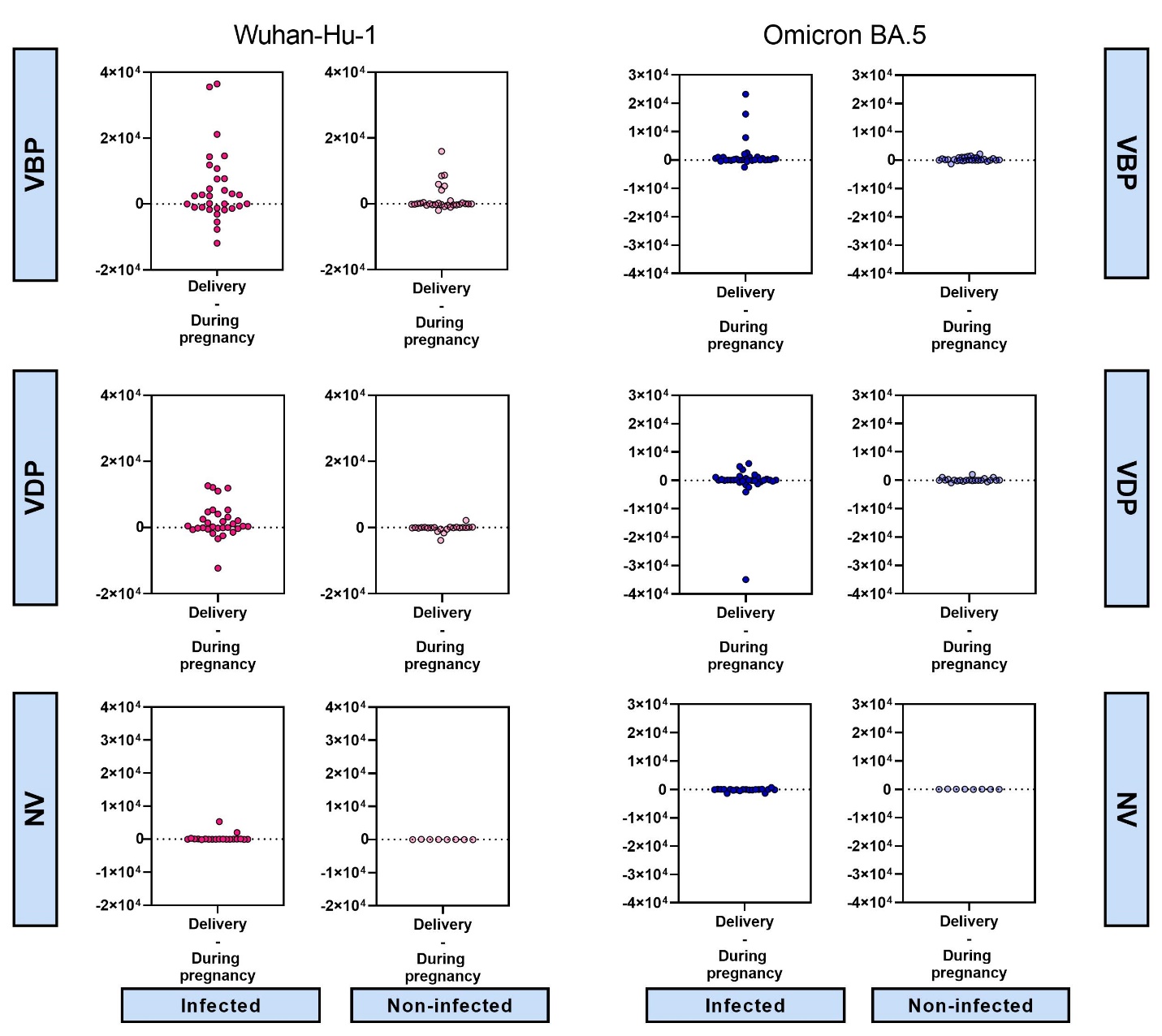
 Supplementary Figure 2.** Difference in neutralizing activity over time. The plots present differences between ND50 values calculated for paired maternal samples collected at different times of pregnancy. Differences were defined as ND50delivery time – ND50during pregnancy.
